# Modelling the COVID-19 Pandemic Effects on Employees’ Health and Performance: A PLS-SEM Mediation Approach

**DOI:** 10.3390/ijerph19031865

**Published:** 2022-02-07

**Authors:** Ion Popa, Simona Cătălina Ștefan, Ana Alexandra Olariu, Ștefan Cătălin Popa, Cătălina Florentina Popa

**Affiliations:** Management Department, The Bucharest University of Economic Studies, 010374 Bucharest, Romania; ion.popa@man.ase.ro (I.P.); anaalexandra.gora@man.ase.ro (A.A.O.); catalin.popa@man.ase.ro (Ș.C.P.); catalina.albu@man.ase.ro (C.F.P.)

**Keywords:** COVID-19, mental well-being, physical well-being, pandemic effects, work performance, stress factors

## Abstract

The COVID-19 pandemic has resulted in the imposition of certain changes in the management of organizations and in the behavior and actions of employees. The purpose of this paper is to investigate the impact of COVID-19 pandemic effects on employees’ health and mental well-being, as well as on their working performance. Moreover, the paper aims to highlight whether health- and work-related stress factors mediate the above relations. For the purpose of data collection, a structured questionnaire was used. The first results of the study showed that the pandemic effects felt by employees did not directly affect their mental and physical well-being. On the other hand, the COVID-19 pandemic effects felt by employees affected their general work performance. The findings of the study may provide a useful perspective for organizations and their employees in order to adopt the most effective measures to minimize the effects generated by the pandemic.

## 1. Introduction

Employees are an important factor, contributing to increasing productivity in and supporting the progress of organizations. As Ariawaty [1] considers, employees are the engine that drives the activities of organizations to achieve goals, and the better they are, the better the performance of that organization, which will ensure success in achieving organizational goals. In the current context, the COVID-19 pandemic does have a serious impact on employees. Negatively and directly or indirectly, all employees in all organizations have been affected by COVID-19, are being affected or will be affected by this pandemic; there are no employees working in any organization that is immune to coronavirus [2]. The COVID-19 pandemic caused, in the last two years, a lot of uncertainty for organizations and their employees, causing them to change the rules relating to working. As the pandemic has spread, social distancing and remote working has become the new normality, still raising concerns and questions about the impact on peoples’ health and work performance among managers and employees.

Thus, it can be seen that the COVID-19 pandemic imposes certain changes in the management of organizations. Investigating the impact of the pandemic on employees’ well-being, as well as on their working performance, has become a key topic during this period. In such emergency situations as the COVID-19 pandemic, employees can react in different ways to stress. In this regard, it is also interesting to study whether health- and work-related stress factors can mediate the relationship between the effects of the pandemic on employees’ health and mental well-being and on their working performance.

Given the novelty of studies that have addressed the effects of the COVID-19 pandemic in an organizational context, few studies have been identified in the literature to investigate how the effects of the pandemic affect the well-being and general performance of employees. For example, the objective of the study conducted by Kawugana and Rabiu [3] was to determine how and the extent to which the pandemic has affected employees’ performance. The results indicated that the COVID-19 pandemic has significantly affected employees’ performance and also their well-being. Another study [4] analyzed the impact of the pandemic on the performance of private companies in 37 countries. The results showed that the COVID-19 pandemic negatively impacted the performance of companies in almost all the countries analyzed, but a stronger effect was observed among firms in developing countries. The main performances affected were financial.

Some studies have focused on specific areas of activity. There are studies [5,6] that have analyzed the COVID-19 pandemic impact on the mental well-being of health professionals. Both studies have shown that the COVID-19 pandemic has led to an increase in the severity of psychological symptoms, especially anxiety and depression. Similarly, the results of the study reported in [7] demonstrated that the COVID-19 pandemic has led not only to an extreme state of anxiety among employees but also to a decrease in job satisfaction and organizational commitment.

The COVID-19 pandemic is affecting both personal and professional lives and people need organizations to keep them informed about security measures and how the crisis is affecting their jobs [8]. Therefore, some studies, e.g., [9], suggest that the COVID-19 pandemic effects should be controlled by support provided at work, as organizational support perceived by employees is associated with positive changes in the well-being of employees at work.

Although there are studies that have analyzed the effects of the COVID-19 pandemic on employee mental well-being and on employee performance, no work has been identified which studies how the COVID-19 pandemic affects employees’ physical health. In addition, the research has not focused on the mediating effect of the level of stress related to health and work on the relationship between the pandemic and the well-being of employees. In addition, to a large extent, research that has analyzed the effects of the COVID-19 pandemic on performance has taken into account organizational performance, not overall employee work performance.

This paper is intended to fill this gap identified in the literature. The aim of this paper is to study how the COVID-19 pandemic effects felt by employees affect their well-being, both physical and mental, and how the COVID-19 pandemic effects felt by employees affect the general work performance of employees. This paper also studies the mediating effect of the level of stress related to health and work in the relationship between the COVID-19 pandemic effects felt by employees and the well-being of employees, both physical and mental, as well as the mediating effect of the level of stress related to health and work in the relationship between the COVID-19 pandemic effects felt by employees and the overall performance of employees.

This paper begins with the specification of the contextual framework and with the analysis of the specialized literature in order to develop the research hypotheses. Then, the research methodology is highlighted and the materials and methods used in this paper are described. Next, the results of the study are presented and the relationships between the effects of the COVID-19 pandemic on employees’ health and mental well-being, as well as on their working performance, are discussed. Furthermore, the extent to which health- and work-related stress factors mediate the above relationships is also emphasized. Finally, the conclusions of this research are presented, highlighting the main implications and limitations of the research.

## 2. Theoretical Backgrounds and Research Hypotheses

### 2.1. The Links between the Effects of the COVID-19 Pandemic and the Physical and Mental Well-Being of Employees

The health of a population depends to a large extent on the socio-economic context, labour markets, welfare systems, public policies, and demographic characteristics of that country, which indicates that there are strong reasons to believe that possible changes in these key factors can also be reflected in the mental well-being of that population [10]. Godderis and Luyten [11] consider that the COVID-19 pandemic will probably lead to a new worldwide economic recession, which is expected to determine different health problems, associated with various aspects of employment. Moreover, these authors believe that a global economic recession will negatively affect the population’s well-being and health, as job uncertainty and unemployment will negatively impact self-esteem, stress, and (ultimately) physical and mental health [11].

COVID-19 pandemic has changed daily routines and brought great challenges in all areas of activity [12,13,14,15,16,17,18]. Moreover, it has implicitly contributed to an increase in fear, anxiety, emotional stress, fatigue, insomnia, sadness, and depression among people [12,19]. According to Qiu et al. [20], the COVID-19 pandemic has caused serious threats to people’s physical health and lives, and it has also triggered different psychological or mental problems, such as panic attacks, anxiety, and depression. Related to this, Li et al. [21] claim that the low predictability and the uncertainty of the COVID-19 pandemic has affected not only people’s mental health, especially in terms of cognition and emotions, but also their physical health. Moreover, Li et al. [21] studied the evolution of emotional and cognitive indicators before and after the COVID-19 pandemic. The results of this study indicated that after the declaration of COVID-19, negative emotional indicators of psychological traits showed an increase in anxiety, depression, and indignation, meanwhile, the positive emotional indicators of psychological traits decreased in Oxford happiness [21]. Furthermore, from a cognition perspective, social risk judgment was higher and life satisfaction was lower after the declaration of COVID-19 [21].

As employees, people felt even more the effects of the COVID-19 pandemic because there were multiple changes in terms of work. In this sense, studies show that this pandemic has had an unprecedented influence on the labor market [22,23,24,25,26,27,28,29,30]. In this regard, Tušl, Brauchli, Kerksieck, and Bauer [31] believe that during the pandemic working conditions have deteriorated and employees are more likely to have mental health problems, such as depression, stress, and anxiety. In crisis situations, such as this pandemic, employees’ understanding increases the success of emergency plans to avoid the spread coronavirus infection and it is extremely important to consider the mental and physical conditions of employees [32]. Ripp, Peccoralo, and Charney [33] believe that these factors of psychological stress require a strong well-being support model for employees. Studies highlights that the level of employees’ physical and mental well-being has tended to decrease during the pandemic, a tendency that may have been caused by emotions like anxiety, fear, or stress, as well as new working conditions imposed as a result of the pandemic [32]. Furthermore, a study by Voydanoff [34] points out that a number of factors, currently known as the COVID-19 pandemic effects, such as income loss, lack of finances, and unemployment, can cause depression and affect the mental health of employees.

Among the effects of the COVID-19 pandemic that have led to changes in work are social distancing and working from home [18,35,36,37,38]. Studies show that social distance and isolation at home have a negative effect on the mental well-being of employees and make them more sedentary and dependent on the use of digital devices, such as phones and laptops [39,40]. Moreover, among employees, increased use of these devices and excessive time spent in front of screens has been negatively correlated with sleep duration and associated with an increase in depressive symptoms, anxiety, headaches, fatigue, and eye symptoms, which shows that these effects of the pandemic have also unbalanced the mental health and physical well-being of employees [39,41]. At the same time, employees who worked from home to meet the requirements of social distancing reported a decrease in physical and mental well-being due to decreased physical activity, increased consumption of unhealthy food, and lack of communication with colleagues [35,40,42,43].

In view of the above, the following research hypotheses can be formulated, regarding the effects of the COVID-19 pandemic on the physical and mental well-being of employees:

**Hypothesis** **1** **(H1).** The COVID-19 pandemic effects felt by employees affected their mental well-being.

**Hypothesis** **2** **(H2).** The COVID-19 pandemic effects felt by employees affected their physical well-being.

### 2.2. The Effects of the COVID-19 Pandemic and Employee Performance

On the other hand, regarding the physical and mental condition of employees, the COVID-19 pandemic effects also influence their work performance. During the COVID-19 pandemic, employees began to become increasingly concerned about safety, the risk of becoming infected with the virus, social exclusion, financial loss, and job insecurity [44], which means that they felt threatened by these effects of the COVID-19 pandemic. In this regard, it may be pointed out that these effects of COVID-19 were the main reason for employees’ stress and the decline in work performance [44]. According to Ince [32], compared with the condition before COVID-19, employee performance during COVID-19 has decreased. Moreover, studying the links between the effects of the COVID-19 pandemic and employees’ performance, Hamid et al. [44] found that the effects of COVID-19 have a significant negative impact on employee work performance. In addition to behavioral changes and the physical and mental condition of employees, the effects of the pandemic, such as social distancing and working from home, also require adjustment of the workplace. Specifically, performing work activities in an inefficient place can have a detrimental effect on the physical and mental well-being of employees, as well as on their overall work performance, leading to their decline [35,45].

Thus, it may be highlighted that the measures taken to prevent the spread of the virus, such as social distancing and working for home, negatively influenced employees’ productivity and, implicitly, that their general work performance decreased during the COVID-19 pandemic. Starting from these aspects, the following research hypothesis can be formulated:

**Hypothesis** **3** **(H3).** The COVID-19 pandemic effects felt by employees affected their general work performance.

### 2.3. The Mediating Role of Stress on the Relationship between COVID-19 Effects and Employee Well-Being

Based on the above, it can be seen that the COVID-19 pandemic has a major impact on the physical and mental well-being of people, especially employees. At the same time, the effects of a pandemic, such as social isolation, financial problems, and the infection of a family member or friend, lead to an increase in general stress levels [19,21]. In this regard, among the main consequences of the stress caused by the effects of the COVID-19 pandemic on the health of employees, especially those in the health system, are the following [19]: influencing the mental health of employees by developing anxiety and depression, developing physical illnesses, such as cardiovascular disease, and diminishing empathy for others. According to Koh and Goh [46], during the SARS outbreak, employees, and especially healthcare workers, worked under great stress and constant fear because in addition to being exposed to the virus, they suffered from fatigue, burnout, and were also exposed to the risk of physical and psychological violence.

In emergencies such as COVID-19, people can react in different ways to stress, and high levels of stress can have serious effects on mental health [47]. Stress associated with uncertainty and unpredictability can have negative consequences on physical well-being and mental health, such as anxiety, depression, and burnout [48]. Moreover, in the COVID-19 pandemic context, the fear of losing one’s job and, consequently, one’s income and job insecurity are major work-related stressors [31,46,49,50] that are associated with poor self-rated health [48]. In addition, the results of the study conducted by Khan et al. [49] emphasize that the fear of economic crisis and unemployment has increased perceptions of job insecurity among employees and has become the main cause of various psychological problems, such as stress, depression, anxiety, and uncertainty during the COVID-19 outbreak. The findings of another study indicate that, during the pandemic, the most common stressors related to work and financial problems and to home matters [51]. At the same time, in the context of this study, these stress factors (stress from work, stress from home, and financial stress), along with other effects of the COVID-19 pandemic, such as fear, helplessness, and dismay, are considered negative indicators of mental health [51].

Opatha [2] identified fifteen fears which an employee may face in the context of the COVID-19 pandemic (such as fear of being infected himself or herself, fear of infecting others, fear of the possibility of having to quarantine, fear of losing their job, etc.) and believes that employees will experience one or two, several or even all of the fifteen identified fears and thus become more stressed. Moreover, Opatha [2] believes that these stressors (fears) also attack the mental health of employees, because too much stress can lead to numerous negative physiological consequences (low immunity, muscle pain, etc.), behavioral consequences (increased alcohol and tobacco consumption), as well as negative psychological consequences (anxiety, lower job satisfaction, lower emotional well-being). Other factors, such as conflicting messages from the authorities, social isolation, large and growing financial losses, and a continuing state of uncertainty, have also been described as other major stressors that cause emotional distress and have a negative effect on the mental health and well-being of employees [31,52,53,54,55].

Starting from the major role of health and work-related stress on the physical and mental well-being of employees, in the context of the effects of the COVID-19 pandemic, the following research hypotheses can be set out:

**Hypothesis** **4** **(H4).** Health- and work-related stress levels mediate the relationship between the COVID-19 pandemic effects felt by employees and their mental well-being.

**Hypothesis** **5** **(H5).** Health- and work-related stress levels mediate the relationship between the COVID-19 pandemic effects felt by employees and their physical well-being.

### 2.4. The Mediating Role of Stress on the Relationship between COVID-19 Effects and Employee Performance

Previous research shows that crises, such as the COVID-19 pandemic, affect not only physical and mental health and well-being but also employee performance [49]. In the current context of the COVID-19 pandemic, the fear of contracting coronavirus has become a stressor for employees, and undoubtedly affects employee performance, whether they are working from home or at the workplace [44]. A number of work-related stress factors, also felt during the pandemic, such as job uncertainty and the threat of unemployment at work, were directly linked to an unexpectedly low level of performance [56]. Starting from the above, the following hypothesis can be formulated:

**Hypothesis** **6** **(H6).** Health- and work-related stress levels mediate the relationship between the COVID-19 pandemic effects felt by employees and their general work performance.

## 3. Materials and Methods

### 3.1. Data collection Procedure

The purpose of this paper was to investigate the impact of COVID-19 pandemic effects on employees’ health and mental well-being, as well as on their working performance. Moreover, the paper aimed to highlight whether health- and work-related stress factors mediate the above relations. In order to achieve the purpose of the research, a survey was designed with a questionnaire as a research tool. Thus, this survey was conducted between November 2020 and January 2021. In the context of this research, this period was a critical one in Romania because the highest number of cases of COVID-19 infection were reported then, which means that the effects of the COVID-19 pandemic could have been felt to an even greater measure. Regarding data collection, the questionnaire was distributed online, via Google Forms, since (i) the target group included employees from all geographical areas in Romania, (ii) the questionnaire could be completed by several respondents at the same time, and (iii) ease of access to the data collected was facilitated. In this way, the collected data was automatically centralized in a database which allowed the data to be processed using statistical methods and software.

### 3.2. Research Population and Sample

The basis of this research was a survey based on a questionnaire that was addressed to persons employed in public or private sector organizations in Romania, regardless of the development region, field of activity, or other particularities of the organization. In this respect, the only criterion for inclusion in the category of potential respondents of this research was that they be employed, otherwise the possibility of completing the questionnaire was blocked. Moreover, potential respondents were asked to provide answers to the questionnaire in relation to their current job and recent conditions. Following the distribution of the questionnaire, a total of 386 questionnaires were completed. Preliminary analyzes showed that there were no cases with suspicious answers or missing data for the variables included in the questionnaire. On the other hand, since 39 responses were from non-employees, they were removed from the database. A total of 347 valid responses were obtained. Thus, for this research, a non-probabilistic sample, including 347 employees from Romania, was used, which consisted of 58.50% women and 41.50% men. Out of these, 67.44% were employed in a private sector organization in Romania and 32.56% worked in a public sector organization. Moreover, the respondents included in the research sample were between 19 and 66 years old, 25.07% of them held a management position, and 74.93% of them were workers without a management position.

### 3.3. Measures

For the purpose of data collection, a structured questionnaire was used. In this research, the hypotheses were operationalized through six constructs, each of them measured by several items, adapted for this purpose from previous studies (see Appendix A). All items were measured on a five-point scale, with the exception of General work Performance. Therefore, the six measurement constructs included in the questionnaire were as follows:

- COVID-19 Effects (C19E). This construct aimed to assess the extent to which employees felt a number of threats/effects of the COVID-19 pandemic. As specific variables, five items were included, such as “technical unemployment”, “salary decreases”, “lifestyle changes imposed by social distancing”, etc.
- Health-Related Stress Factors (HRSFs). Two items were included in this category:” fear of becoming infected with coronavirus” and “fear of infecting others”. These stressors indicated the extent to which employees felt stressed about their health and the health of those around them.
- Work-Related Stress Factors (WRSFs) included five items, such as “The fear of losing a job”, ”The fear of working from home for a long time”. This category is important because it highlights the main work-related fears that employees felt during the pandemic and which were a stress factor for them.
- Mental Well-Being (MWB). The aim of this construct was to determine the mental or psychological experience felt by those who worked during the COVID-19 pandemic. Seven items were included in this category, for example: ”Feeling nervous”, “Feeling hopeless”, ”Feeling impatient or irritable”.
- Physical Well-Being (PWB). Seven items, which highlight the extent to which employees felt a number of conditions that impaired their physical well-being, were included in this construct. Some of the specific aspects used in the research were: “Feeling tired or having low energy”, “Headaches”, “Trouble sleeping”, “Fever or cold symptoms”, ”Muscle soreness”.
- General Work Performance (GWP). Given the fact that potential respondents could be employed in any organization in Romania, overall work performance was assessed by means of one item adopted from the WHO Health and Work Performance Questionnaire (HPQ) [57,58]. This item was chosen because it can be used regardless of the particularities of the organization to assess the overall performance of employees, but also because it is frequently used in this respect [59,60,61,62,63,64,65]. Therefore, respondents rated their recent overall work performance using a 10-point scale, ranging from 1 (worst possible work performance that a person could demonstrate in this job) to 10 (top work performance on the job).

The six measurement constructs, described above, as well as their corresponding references are presented in Table 1.

As for the questions included in the questionnaire, these were closed questions. A 5-point numerical scale was used to measure them, where 1 meant that employees were not described at all by a particular statement, and 5 meant that the statement described their situation to a very large extent. In order to carry out the survey properly and for the accuracy of the data collection, the questionnaire was preceded by initial information through which respondents were informed of the purpose of the research; it was stated that the answers provided would be used exclusively for scientific purposes and that the quality of the study depends decisively on the seriousness of the answers provided. At the same time, it was pointed out that the survey was part of a research project, and the potential respondents were informed that the answers provided in the questionnaire would be confidential and that their participation in this survey was voluntary. At the outset, the time required to complete the questionnaire was also mentioned. A closed-ended question was also included at the beginning of the questionnaire, in which respondents agreed to participate in this research.

### 3.4. Model Specification

The purpose of this paper is to investigate the impact of COVID-19 pandemic effects on employees’ health and mental well-being, as well as on their working performance. We also intend to highlight if health- and work-related stress factors mediate the above relations.

In this study, structural equation modeling was employed to represent the proposed relationships and to test the research hypothesis. As stated by Henseler and Sarstedt [66], the PLS-SEM models are defined by two sets of linear equations: the other (measurement) model, which specifies the relationships between the unobserved constructs (latent variables) and their indicators (manifest variables), and the inner (structural) model, which refers to the relationships between unobserved constructs (latent variables). Therefore, in the structural model one exogenous construct was included: COVID-19 Effects (C19E); and five endogenous ones: Health-Related Stress Factors (HRSFs), Work-Related Stress Factors (WRSFs), Mental Well-Being (MWB), Physical Well-Being (PWB), and General Work Performance (GWP).

The analysis, performed with the SmartPLS application, version 3.3.3(GmbH, Bönningstedt, Germany) [67], included both the evaluation of the measurement and structural model as well as the direct and indirect (mediated) relationships.

## 4. Results

### 4.1. Measurement Model

Evaluation of the PLS-SEM measurement model included [68] the examination of the indicator and construct reliability and convergent and discriminant validity. In terms of reliability (see Table 2), the factor loadings, Cronbach Alpha coefficients, and composite reliability (CR) have values above the recommended thresholds of 0.708, 0.7, and 0.7, respectively [68,69]. To achieve these results, from the initial physical well-being scale, two items (PWB8 and PWB9) were excluded due to their low factor loading. Therefore, evidence can be considered to support the reliability of measurement models.

Furthermore, the convergent validity was evaluated by computing the average variance extracted (AVE) for each construct, all of which were found to be higher than the recommended value of 0.5 [70], and the factor loadings, while the discriminate validity was assessed using the Fornell–Larcker [71] criterion and Henseler et al.’s [72] new criterion of heterotrait–monotrait ratio of correlations (HTMT). First, as may be seen in Table 3 and Table 4, the AVE of each construct is greater than the squared correlation with all others constructs, while the HTMT ratios range between 0.334 and 0.742, therefore not exceeding Henseler et al.’s [72] thresholds of 0.90 or 0.85. On the above evaluation, the convergent and discriminant validity of all constructs of the measurement model was established.

### 4.2. Structural Model

**Figure 1:**
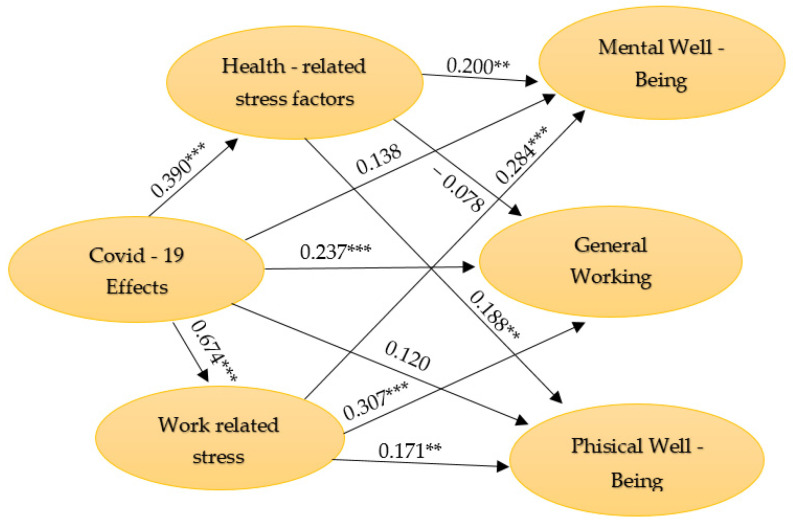
The structural model. Source: authors’ computation with SmartPls 3.3.3 [67]. *** *p* < 0.001; ** *p* < 0.01.

In terms of structural model evaluation, first, the possible collinearity issues between the predictors were excluded, since all the values were under the maximum debated value of 5 [68]. Furthermore, the endogenous constructs’ coefficients of determination $\left(R^{2}\right)$ indicated that 15.2% of the variance in Health-Related Stress Factors and 45.4% in Work-Related Stress Factors may be explained by COVID-19 Effects. Moreover, COVID-19 Effects and Stress Factors may determine 23.8% of variance in employees’ Mental Well-Being, 16.4% in their Physical Well-Being and 4.4% in General Working Performance. Figure 1 presents the structural model.

### 4.3. Effects of the COVID-19 Pandemic 

To validate the first three research hypotheses, the relevance and significance of path coefficients of the direct relationships of C19E effects were evaluated. In Table 5 are included the path coefficients (*β*) and their associated t statistics. The significant path coefficient between C19E and GWP (β=−0.237;p<0.001) gives support for hypothesis 1. Therefore, the COVID-19 pandemic effects felt by employees affected their general work performance, such that the more intensely they felt threatened by COVID-19 effects, such as cessation of activity at work, salary decreases, technical unemployment, changes in their lifestyle due to changes in interpersonal relationships imposed by isolation, physical distancing, or changing attitudes of people around them, the lower their work performance.

On the other hand, the path coefficients between C19E and MWB (β=0.137;ns) and PWB (β=0.120;ns), respectively, cannot support hypotheses 1 and 2. Therefore, the COVID-19 pandemic effects felt by employees did not directly affect their mental and physical well-being.

### 4.4. Mediating Role of Stress Factors

Besides the direct effects of the COVID-19 pandemic, the indirect effects mediated by Health- and Work-Related Stress Factors were investigated by computing the bias-corrected confidence intervals of indirect effects through a bootstrapping procedure with 5000 sub-samples (Table 6).

Considering the mediation effect of the HRSFs and WRSFs on the C19E -> MWB and C19E -> PWB relationships, both the total and specific indirect effects were found to be significant, while the direct ones were not. These results support the total mediation effects and support hypotheses 4 (a and b) and 5 (a and b). Therefore, the more intensely employees feel threatened by COVID-19 effects, such as cessation of activity at work, salary decreases, technical unemployment, changes in their lifestyle as a result of changing interpersonal relationships imposed by isolation, physical distancing, or changing attitudes of people around them, the more they will be stressed by the fear of falling sick themselves or of someone they love falling sick, of losing their jobs, of distancing from those close to them, of working from home for a long time, or of going into technical unemployment, and, in turn, their mental and physical well-being will deteriorate.

In contrast to the effects with negative connotations of the mediating role of stressors on the health of employees, effects with positive connotations on their level of work performance could also be observed. Thus, considering the positive indirect effect of COVID-19 effects on GWP mediated by Work-Related Stress Factors (β=0.207;t=4.032;95BCI [0.111, 0.313]) and also the negative direct effect, we may conclude that Work-Related Stress Factors concurrently mediated the C19E -> GWP relationship, thus supporting hypothesis 6b. Therefore, the more intensely employees feel stressed by the fear of decreasing income, losing their jobs, distancing from those close to them, working from home for a long time, or going into technical unemployment, the better they will cope with COVID-19 effects, thus decreasing the negative impact on their work performance. On the other hand, Health-Related Stress Factors do not have such a mediating effect and hypothesis 6a could not be confirmed by the empirical results.

## 5. Discussion

There are few studies in the literature that have investigated how the effects of the COVID-19 pandemic affect the physical and mental well-being of employees and also their general work performance. According to the results of the present study, the COVID-19 pandemic effects felt by employees affected their general work performance. The same results were obtained following the study carried out by Kawugana and Mohammed [3], who demonstrated that the COVID-19 pandemic has significantly affected employees’ performance, and also the study carried out by Narayanamurthy and Tortorella [73], who demonstrated that COVID-19′s work implications (i.e., the home office work environment, job insecurity, and virtual connection) do impact employee’s performance, although not to the same extent.

The study conducted by Kawugana and Mohammed [3] also analyzed the extent to which the COVID-19 pandemic affected the well-being felt by employees. The results showed that the COVID-19 pandemic has significantly affected their well-being. Contrarily, the hypotheses of the present study, according to which the COVID-19 pandemic effects felt by employees affected their mental and physical well-being, could not be validated. In contrast with the results of the present study, there are a number of previous studies [5,6,7,74] that have analyzed the impact of the COVID-19 pandemic on mental well-being of health professionals and which have demonstrated that the COVID-19 pandemic has led to an extreme state of anxiety and depression among employees. In the same vein, the present paper does not agree with the study conducted by Lin et al. [75], the results of which show that the novelty of the COVID-19 pandemic is positively related to the professional insecurity perceived by employees, which in turn is positively related to their emotional exhaustion. Conducting a survey on healthcare and other workers, Evanoff et al. [76] demonstrated that among all workers anxiety, depression and high work exhaustion were independently associated with community or clinical exposure to COVID-19. This effect is felt not only by employees but also by students. The results of the study conducted by Son, Hegde, Smith, Wang, and Sasangohar [77] have shown that there is a high level of stress and anxiety among students as a result of the COVID-19 pandemic.

Therefore, contrary to the results of the present study, according to which the COVID-19 pandemic effects felt by employees did not affect their physical and mental well-being, the literature largely demonstrated that the COVID-19 pandemic negatively affected the well-being of employees in terms of workplace relationships and work–life balance [78]. At the same time, the literature demonstrated that the COVID-19 pandemic has alarming implications for collective health and emotional functioning of the individual [52]. According to Vizheh et al. [79], during the COVID-19 pandemic, healthcare workers have faced aggravated psychological pressure and even mental illness.

The hypotheses that studied the mediating role of stress levels related to health and work on the relationship between the COVID-19 pandemic effects felt by employees and their mental and physical well-being, and also on the relationship between the COVID-19 pandemic effects felt by employees and their general work performance, cannot be compared with other studies because no papers have been identified in the literature to focus on these mediation relationships.

## 6. Conclusions

The aim of this paper was to study how the COVID-19 pandemic effects felt by employees affected their physical and mental well-being and how these effects impact on the general work performance of employees. This study also investigated the effects mediated by health- and work-related stress factors, considering the relationship between the COVID-19 pandemic effects felt by employees and their well-being, both mental and physical, and also considered the relationship between the COVID-19 pandemic effects felt by employees and their general work performance.

The research tool used for data collection was the structured questionnaire and the program used for data interpretation and analysis was SmartPls 3.3.3. The hypotheses were operationalized through six constructs, namely, COVID-19 effects, Health-Related Stress Factors, Work-Related Stress Factors, Mental Well-Being, Physical Well-Being, and General Work Performance.

The first results of the study showed that the COVID-19 pandemic effects felt by employees did not directly affect their mental and physical well-being. On the other hand, the COVID-19 pandemic effects felt by employees affected their general work performance. Therefore, the more intensely they felt threatened by COVID-19 effects, the more their work performance decreased. Considering the mediating role of stress factors, the results of the study showed that health- and work-related stress levels mediated the relationship between the COVID-19 pandemic effects felt by employees and their well-being, both mental and physical. In this regard, the more intensely employees feel threatened by COVID-19 effects, the more their mental and physical well-being will deteriorate. On the other hand, health-related stress factors do not mediate the relationship between the COVID-19 pandemic effects felt by employees and their general work performance.

The results of this study may lead to an awareness among employees that the COVID-19 pandemic is an external factor that affects not only the economy and organizations as a whole but also the well-being of employees and their performance at work. However, the results of this study, according to which the COVID-19 pandemic effects felt by employees, does not affect their mental and physical well-being, can be considered unexpected, taking into account the studies identified in the literature, which have shown the opposite. Moreover, the usefulness of the results of this study stems from the fact that organizations can focus significantly on human resource management and use a series of strategies to minimize or eliminate the impact of the COVID-19 pandemic on employee well-being and outcomes. At the same time, the study may provide insight into future research that may consider how the COVID-19 pandemic may influence other factors, such as employee motivation, certain employee characteristics, or specific aspects of mental well-being (e.g., depression, anxiety, panic disorder).

Although the results of this study may have important theoretical and practical implications, certain limitations should also be addressed. One limitation of this research is the fact that the number of existing studies in this field are relatively small, due to the fact that the COVID-19 pandemic is a new topic that has not been widely addressed so far. Another limitation of this research may be that the COVID-19 pandemic effects felt by employees can affect a multitude of other factors, not just the mental and physical well-being of employees and overall work performance. Moreover, overall performance is extremely comprehensive and thus it is not possible to know exactly which components of work performance are affected by the COVID-19 pandemic. Overall performance includes aspects such as productivity and quality of work, employee skills, the interpersonal qualities of employees, and the degree of achievement of objectives. On the other hand, another limitation of this research is related to the fact that the study was not a longitudinal one, the time period chosen for data collection being November 2020–January 2021. In this regard, there might be a change of perceptions of COVID-19 at the end of 2020 compared to the beginning of 2021 which could affect the findings, since the study measures individual perceptions. All of these limitations may represent directions for future research.

## Figures and Tables

**Table 1:** Research measures.

Construct	Items	Variables	References
COVID-19 Effects (C19E)	5	C19E1–C19E5	[31,49,50]
Health-Related Stress Factors (HRSFs)	2	STR1–STR2	[2,46,50]
Work-Related Stress Factors (WRSFs)	5	STR3–STR7	[2,31,46,49]
Mental Well-Being (MWB)	7	MWB1–MWB7	[57,58]
Physical Well-Being (PWB)	7	PWB1–PWB7	[57,58]
General Work Performance (GWP)	1	GWP	[57,58,59,60,61,62,63,64,65]

**Table 2:** Construct reliability and validity.

Latent Construct (Reflective/Formative)	Items	Loadings	*t*	α	Rho_A	CR	AVE
COVID-19 Effects (C19E) (reflective)	C19E1	0.857	45.196	0.892	0.893	0.921	0.699
	C19E2	0.880	60.359				
	C19E3	0.830	37.065				
	C19E4	0.802	31.530				
	C19E5	0.809	33.797				
Health-Related Stress Factors (HRSFs) (reflective)	STR1	0.923	88.282	0.793	0.805	0.906	0.828
	STR2	0.896	59.036				
Work-Related Stress Factors (WRSFs) (reflective)	STR3	0.874	56.395	0.912	0.915	0.934	0.740
	STR4	0.890	65.396				
	STR5	0.805	31.891				
	STR6	0.848	41.756				
	STR7	0.881	58.225				
Mental Well-Being (MWB)	MWB1	0.815	31.068	0.946	0.947	0.956	0.755
(reflective)	MWB2	0.844	38.085				
	MWB3	0.899	66.034				
	MWB4	0.883	63.107				
	MWB5	0.880	42.992				
	MWB6	0.903	82.098				
	MWB7	0.856	42.036				
Physical Well-Being (PWB)	PWB1	0.797	29.288	0.925	0.928	0.940	0.689
(reflective)	PWB2	0.848	48.945				
	PWB3	0.834	37.196				
	PWB4	0.832	40.398				
	PWB5	0.832	41.688				
	PWB6	0.841	38.659				
	PWB7	0.829	34.751				

α, Cronbach’s Alpha; AVE, Average variance extracted; and CR, Composite reliability. Source: authors’ computation with SmartPls 3.3.3 [67].

**Table 3:** Fornell–Larcker Criterion.

Constructs	(1)	(2)	(3)	(4)	(5)
COVID-19 Effects	0.836				
Health-Related Stress Factors	0.390	0.910			
Mental Well-Being	0.407	0.425	0.869		
Phisical Well-Being	0.309	0.338	0.679	0.830	
Work-Related Stress Factors	0.674	0.604	0.498	0.366	0.860

Source: authors’ computation with SmartPls 3.3.3 [67].

**Table 4:** Heterotrait–Monotrait Ratio (HTMT).

Constructs	(1)	(2)	(3)	(4)	(5)
COVID-19 Effects					
Health-Related Stress Factors	0.456				
Mental Well-Being	0.437	0.489			
Phisical Well-Being	0.334	0.392	0.726		
Work-Related Stress Factors	0.742	0.712	0.534	0.395	

Source: authors’ computation with SmartPls 3.3.3 [67].

**Table 5:** Testing for direct effects.

Relationship	*β*	SE	t	95% BC CI		Hypotheses	Decision
				CI_low_	CI_high_		
C19E -> MWB	0.137	0.071	1.936	−0.002	0.278	H1	Not supported
C19E -> PWB	0.120	0.063	1.897	−0.009	0.242	H2	Not supported
C19E -> GWP	−0.237 ***	0.071	3.351	−0.370	−0.093	H3	Supported

Note: *** *p* < 0.001; *β*, Standard coefficients; SE, Standard error; BC CI, Bias-corrected confidence intervals. Source: authors’ computation with SmartPls 3.3.3 [67].

**Table 6:** Testing for indirect effects.

Relationship	*β*	SE	t	95% BC CI		Hypothesis	Decision
				CI_low_	CI_high_		
C19E -> MWB ^a^	0.407 ***	0.051	7.971	0.304	0.500		
C19E -> MWB ^b^	0.137	0.071	1.936	−0.002	0.278		
C19E -> MWB ^c^	0.270 ***	0.048	5.647	0.180	0.367		
C19E -> HRSFs -> MWB	0.078 **	0.028	2.770	0.027	0.138	H4a	Supported
C19E -> WRSFs -> MWB	0.192 ***	0.056	3.427	0.086	0.308	H4b	Supported
C19E -> PWB ^a^	0.309 ***	0.053	5.806	0.198	0.406		
C19E -> PWB ^b^	0.120	0.063	1.897	−0.009	0.242		
C19E -> PWB ^c^	0.188 ***	0.046	4.068	0.100	0.280		
C19E -> HRSFs -> PWB	0.074 **	0.028	2.610	0.022	0.133	H5a	Supported
C19E -> WRSFs -> PWB	0.115 *	0.053	2.165	0.010	0.219	H5b	Supported
C19E -> GWP ^a^	−0.061	0.059	1.036	−0.177	0.051		
C19E -> GWP ^b^	−0.237 ***	0.071	3.351	−0.370	−0.093		
C19E -> GWP ^c^	0.176 ***	0.044	3.991	0.089	0.261		
C19E -> HRSFs -> GWP	−0.031	0.026	1.166	−0.086	0.018	H6a	Not supported
C19E -> WRSFs -> GWP	0.207 ***	0.051	4.032	0.111	0.313	H6b	Supported

Note: ^a^ Total effect; ^b^ Direct effect; ^c^ Total indirect effect; 95% BC CI, Bias-corrected confidence intervals; *** *p* < 0.001; ** *p* < 0.01; * *p* < 0.05; *β*, Standardized coefficients; SE, Standard error. Source: authors’ computation with SmartPls 3.3.3 [67].

## Data Availability

The data are not publicly available due to confidentiality reasons.

## Appendix A

**Table:**

Construct	Items	Variables	References
COVID-19 Effects (C19E)	To what extent did you feel the following during the COVID-19 pandemic? 1. Cessation of activity at work 2. Wage decrease 3. Technical unemployment 4. Lifestyle change through interpersonal relationships, change due to isolation, social distance, etc. 5. Attitude change of people around	C19E1–C19E5	[31,49,50]
Health-Related Stress Factors (HRSFs)	To what extent have you been stressed by the following factors lately? 1. Fear of getting sick/ill 2. Fear of someone close getting sick/ill	STR1–STR2	[2,46,50]
Work-Related Stress Factors (WRSFs)	To what extent have you been stressed by the following factors lately? 3. Fear of a decrease in your income 4. Fear of job loss 5. Fear of getting apart from close ones 6. Fear of staying home for a long period of time 7. Fear of starting technical unemployment	STR3–STR7	[2,31,46,49]
Mental Well-Being (MWB)	To what extent do you think the following statements describe your recent experience? 1. I felt sad 2. I felt nervous 3. I felt restless or fidgety 4. I felt hopeless 5. I felt everything was an effort 6. I felt unable to relax 7. I felt impatient or irritable	MWB1–MWB7	[57,58]
Physical Well-Being (PH_WB)	To what extent do you think you faced the following situations during the pandemic? 1. Feeling dizzy 2. Feeling tired or having low energy 3. Trouble sleeping 4. Headache 5. Back or neck pain 6. Arms, legs, or joints pain 7. Muscle soreness 8. Cough or sore throat * 9. Fever, chills, or other cold symptoms *	PWB1–PWB9	[57,58]
General Work Performance (GWP)	1. On a scale of 1 to 10, how would you rate your overall job performance during the last period of time (where 1 means worst performance and 10 means top performance)?	GWP	[57,58,59,60,61,62,63,64,65]

* Items not included in the model due to low loadings.
